# Incidence and Risk Factors for Developing Dengue-Associated Hemophagocytic Lymphohistiocytosis in Puerto Rico, 2008 - 2013

**DOI:** 10.1371/journal.pntd.0004939

**Published:** 2016-08-24

**Authors:** Esther M. Ellis, Tyler M. Sharp, Janice Pérez-Padilla, Liza González, B. Katherine Poole-Smith, Emmaculate Lebo, Charlotte Baker, Mark J. Delorey, Brenda Torres-Velasquez, Eduardo Ochoa, Brenda Rivera-Garcia, Hector Díaz-Pinto, Luis Clavell, Anabel Puig-Ramos, Gritta E. Janka, Kay M. Tomashek

**Affiliations:** 1 Centers for Disease Control and Prevention, Dengue Branch, San Juan, Puerto Rico; 2 Centers for Disease Control and Prevention, Division of Scientific Education and Professional Development, Atlanta, Georgia, United States of America; 3 Ponce Health Sciences University, Ponce, Puerto Rico; 4 Centers for Disease Control and Prevention, Division of Vector-borne Diseases, Fort Collins, Colorado, United States of America; 5 Puerto Rico Children’s Hospital, Bayamon, Puerto Rico; 6 Puerto Rico Department of Health, San Juan, Puerto Rico; 7 San Jorge Children’s Hospital, San Juan, Puerto Rico; 8 University of Puerto Rico, School of Medicine, San Juan, Puerto Rico; 9 University Medical Center, Hamburg, Germany; Pediatric Dengue Vaccine Initiative, UNITED STATES

## Abstract

**Background:**

Hemophagocytic lymphohistiocytosis (HLH) is a rare, potentially fatal disorder characterized by fever, pancytopenia, hepatosplenomegaly, and increased serum ferritin. HLH is being increasingly reported as a complication of dengue, a common tropical acute febrile illness.

**Methodology/Principal Findings:**

After a cluster of pediatric dengue-associated HLH patients was identified during the 2012–2013 dengue epidemic in Puerto Rico, active surveillance and a case-control investigation was conducted at four referral hospitals to determine the incidence of HLH in children and identify risk factors for HLH following dengue. Patients with dengue-associated HLH (cases) were matched by month of illness onset and admission hospital to dengue patients that did not develop HLH (controls). During 2008–2013, a total of 33 HLH patients were identified, of which 22 (67%) were associated with dengue and 1 died (dengue-associated HLH case-fatality rate: 4.5%). Two patients with dengue-associated HLH had illness onset in 2009, none had illness onset during the 2010 dengue epidemic, and 20 had illness onset during the 2012–2013 epidemic. Frequency of infection with either dengue virus (DENV)-1 or DENV-4 did not differ between cases and controls. Cases were younger than controls (median age: 1 vs. 13 years, p < 0.01), were hospitalized longer (18 vs. 5 days, p < 0.01), and were admitted more frequently to pediatric intensive care units (100% vs. 16%, p < 0.01). Cases had co-infection (18.2% vs. 4.5%, p = 0.04), recent influenza-like illness (54.5% vs. 25.0%, p = 0.01), and longer duration of fever (7 vs. 5 days; p < 0.01). Cases were more likely to have lymphadenopathy, hepatomegaly, splenomegaly, anemia, and elevated liver transaminases (p ≤ 0.02).

**Conclusions/Significance:**

During this cluster of dengue-associated HLH cases that was temporally associated with the 2012–2013 epidemic, most patients with dengue-associated HLH were infants and had higher morbidity than dengue inpatients. Physicians throughout the tropics should be aware of HLH as a potential complication of dengue, particularly in patients with anemia and severe liver injury.

## Author Summary

Hemophagocytic lymphohistiocytosis (HLH) is a rare, potentially fatal medical condition that can occur after a patient has an infection. While HLH is most commonly associated with Epstein-Barr virus infections, it has been reported as a complication of dengue, a common mosquito-borne, acute febrile illness. After a cluster of pediatric dengue-associated HLH patients was identified in Puerto Rico, active surveillance and a case-control investigation was conducted to determine the rate of HLH in children and identify risk factors for HLH following dengue. During 2008–2013, a total of 33 HLH patients were identified, of which 22 (67%) were associated with dengue and 1 died (dengue-associated HLH case-fatality rate: 4.5%). Most (91%) dengue-associated HLH patients had illness onset during the 2012–2013 epidemic, however, HLH was not found to be associated with a particular type of dengue virus. Dengue-associated HLH cases were younger than dengue inpatient controls, were hospitalized longer, and were admitted more frequently to the pediatric intensive care unit. Cases had longer duration of fever, and were more likely to have anemia, hepatomegaly and elevated liver transaminases than controls. Physicians in the tropics should be aware that HLH may complicate dengue, and they should evaluate dengue patients who develop anemia and severe liver injury.

## Introduction

Hemophagocytic lymphohistiocytosis (HLH) is a rare, potentially fatal hematologic disorder characterized by hyperinflammation, uncontrolled proliferation of activated lymphocytes, prolonged fever, pancytopenia, jaundice, and hepatosplenomegaly [1]. The etiology of HLH can be familial (primary) or acquired (secondary). Diagnostic testing is available for five genes, *PRF1*, *MUNC13-4*, *STX11*, *RAB27A* and *STXBP2*, which are known to cause familial HLH, and mutations in these genes usually manifest within the first two years of life. The incidence of familial HLH is estimated to be 1.2 cases per 100,000 population per year [1, 2]. Approximately 70% of HLH cases present during infancy, although juveniles and adults presenting with familial HLH have also been reported [1]. Treatment for familial HLH consists of hematopoietic stem cell transplantation to replace defective immune effector cells; if untreated, familial HLH is invariably fatal [3]. Acquired HLH is a result of strong immunological activation associated with infection, malignancies, or autoimmune disorders, and is more common than primary HLH [1]. The diagnosis of acquired HLH is based upon completion of the HLH clinical case definition [1], and treatment consists of administration of high-dose corticosteroids, intravenous immunoglobulin (IVIG), and cyclosporine with or without etoposide to dampen the immune response [1]. Epstein-Barr virus (EBV) is the most common infectious agent associated with development of HLH [1, 4].

Dengue is an acute febrile illness caused by any of four mosquito-borne dengue viruses (DENV-1–4), and is characterized by fever, myalgia, arthralgia, eye pain, headache, rash, and leukopenia [5]. Dengue is endemic throughout the tropics and subtropics, where an estimated 390 million infections occurred in 2010 [6]. Roughly 5% of clinically-apparent dengue cases will progress to severe dengue, which is characterized by plasma leakage leading to effusions, respiratory difficulty, hypovolemic shock, and hemorrhage [5]. Recent investigations have demonstrated that higher serum ferritin levels are associated with dengue as compared to other acute febrile illnesses, and that degree of elevation of ferritin is associated with disease severity and a pro-inflammatory cytokine profile [7, 8]. Similar to hemophagocytic syndromes, infection with either DENV or EBV results in a genetic immune response profile that is associated with uncontrolled inflammatory responses [9], and these immunologic pathways may be common to those that lead to the development of acquired HLH. A total of 74 dengue-associated pediatric and adult HLH cases have been described since 1966 [10–37], in which the cumulative case-fatality rate (CFR) was 9.5%. However, risk factors associated with increased risk for dengue patients to develop HLH, such as age, early clinical characteristics, and infecting DENV, have not been previously investigated.

Dengue has been endemic in the United States Caribbean territory of Puerto Rico since the late 1960s [38, 39], where epidemics occur roughly every 3–5 years. During the most recent epidemic in Puerto Rico during 2012–2013, a total of 29,386 suspected dengue cases were reported via passive surveillance from throughout the island, of which 54% had laboratory evidence of acute DENV infection (Puerto Rico Department of Health [PRDH]). Dengue-associated HLH was first documented in Puerto Rico in 2010 in a 10-month-old patient with severe dengue [19]. However, dengue-associated HLH may be under-recognized in Puerto Rico and other dengue-endemic areas due to overlapping signs and symptoms of HLH and dengue (e.g., fever, hepatosplenomegaly, leukopenia, and thrombocytopenia). Identification of dengue patients with or at risk of developing HLH may be enabled by detection of markedly elevated serum ferritin [22].

In December 2012, six cases of HLH were reported to PRDH from Puerto Rico Children’s Hospital. The majority of these cases occurred during a two-month period (November–December 2012) and had initially been admitted due to dengue. PRDH and CDC worked with local clinicians to investigate the cluster of HLH cases associated with dengue to estimate the incidence of HLH in Puerto Rico and identify risk factors associated with developing HLH following dengue.

## Methods

### Ethics statement

The protocol for this investigation was reviewed by human subjects’ research advisors at the CDC and all hospitals included in the investigation, and was deemed to be a public health intervention and not research. As such, full IRB review was not required.

### Investigation population

Puerto Rico comprises 78 municipalities on three populated islands (9,104 km^2^), of which the total population in 2010 was 3,725,789 and the pediatric population was 903,295 [40]. Roughly half of the residents of Puerto Rico live in the capital, San Juan, and the surrounding metropolitan area. The population included in this investigation included all patients that presented or were transferred to four referral hospitals in the San Juan metropolitan area (Puerto Rico Children’s Hospital, San Jorge Children’s Hospital, San Francisco Hospital, and Hospital HIMA-San Pablo Bayamon) during January 2008 through June 2013.

### Active HLH surveillance

To identify patients with HLH, only patients aged ≤18 years were included since only pediatric HLH patients had been initially reported and two of the four facilities included in the investigation were pediatric hospitals. Hospital billing and discharge diagnoses at each of the four facilities were queried for patients with the following criteria: 1) discharge diagnosis of HLH, hemophagocytic syndrome, macrophage activation syndrome (MAS), pancytopenia, hepatomegaly, splenomegaly, or hepatosplenomegaly; 2) quantitated serum ferritin; or 3) bone marrow biopsy or aspirate performed. Patients meeting any of these criteria were defined as “potential HLH patients”. Medical records of potential HLH patients were retrieved and reviewed to determine if the patient met the HLH clinical case definition (i.e., establishment of at least five out of eight diagnostic criteria including: fever; splenomegaly; cytopenia; hypertriglyceridemia and/or hypofibrinogenemia; hemophagocytosis observed in bone marrow, cerebral spinal fluid, or lymph nodes; decreased or absent NK-cell activity; serum ferritin ≥500 μg/L; and serum IL-2 receptor ≥2,400 units/L) [1]. Patients meeting this case definition were defined as an “HLH patient”. All HLH patients with a diagnosis of leukemia or another cancer of the immune system were excluded from further review. A “familial HLH patient” was defined by a positive genetic HLH test. An “acquired HLH patient” was defined by diagnosis of an infectious disease or exacerbation of a chronic medical condition known to be associated with HLH (e.g., juvenile arthritis) within two weeks of onset of HLH. All identified HLH patients were subsequently queried in the passive dengue surveillance system (PDSS) to determine if they also had tested laboratory-positive for dengue during the illness for which they sought medical care. If so, they were defined as a “dengue-associated HLH patient”, including if there was diagnostic evidence of infection with an additional etiologic agent. For all dengue-associated HLH patients, data including sociodemographic characteristics, previous medical history, clinical signs and symptoms, and laboratory test results were abstracted and anonymously recorded from patients’ medical records.

### Laboratory diagnostics

Dengue diagnostic testing had previously been completed for all individuals involved in this investigation as part of routine dengue surveillance as previously described [38]. In brief, specimens from suspected dengue cases were: a) reported to PDSS and tested to detect DENV nucleic acid and/or anti-DENV IgM antibody by real-time reverse transcription polymerase chain reaction (rRT-PCR) [41] and/or ELISA (InBios International, Inc., Seattle, WA), respectively, depending on the day post-illness onset of specimen collection; or b) sent to a private diagnostic laboratory for testing by anti-DENV IgM ELISA. Specimens that tested positive by rRT-PCR or IgM ELISA were defined as a laboratory-positive dengue case. Clinical dengue case definitions employed followed either 2009 [5] or 1997 [42] World Health Organization (WHO) guidelines. In brief, patients with dengue were defined by presence of fever plus two or more of following: nausea, vomiting, rash, aches and pains, tourniquet test positive, leucopenia (i.e., a white cell count <5.0×10^9^ cells/L), or any warning sign. Warning signs included severe abdominal pain, persistent vomiting, mucosal bleed, lethargy or restlessness, liver enlargement ≥2 cm, and concurrent rise in hematocrit with rapid decrease in platelet count to <100,000/mm^3^. Patients with severe dengue were defined by presence of any of the following: 1) plasma leakage leading to shock or fluid accumulation resulting in acute respiratory distress; 2) severe bleeding with hemodynamic instability requiring fluid replacement and/or blood transfusion, or an intracranial bleed; or 3) severe organ impairment such as acute liver failure, myocarditis, or encephalitis. Patients with dengue hemorrhagic fever (DHF) were defined by presence or history of fever, hemorrhagic manifestations, thrombocytopenia (i.e., platelet count ≤100,000/mm^3^), and plasma leakage.

ELISA performed at CDC was used to determine ferritin and interleukin 2 receptor (IL-2R) concentrations in serum specimens from cases and controls. Serum specimens were diluted either serially 4-fold (1:16–1:1024) prior to testing for human ferritin (MyBioSource, San Diego, CA), or 1:4 and 1:8 prior to testing for IL-2R with the Quantikine ELISA (R&D Systems, Minneapolis, MN). Testing and analysis was performed according to the manufacturer’s instructions. Briefly, specimen concentrations were interpolated from a four-parameter logistics curve of standard concentrations and corrected for the dilution factor. Results were reported as the average of duplicates.

### Statistical analysis

Each identified dengue-affiliated HLH patient (i.e., cases) was matched to four laboratory-positive dengue patients (i.e., controls) by month of onset of illness and site of hospitalization. Data were analyzed using R v3.2.0. For univariate analyses, disease classification was regressed on a suspected risk factor using exact conditional logistic regression. Age was included in each model as a main effect but not interacted with the suspected risk factor. There were insufficient data to perform multiple regression analysis with more than one risk factor at a time. Univariate confidence intervals and p-values were not adjusted for multiple comparisons.

## Results

### Identification of HLH patients

After reviewing the medical records of 694 potential HLH patients that presented during January 2008 through June 2013 to the four hospitals included in the investigation, a total of 33 (4.8%) patients were identified that met the HLH clinical case definition (0.66 HLH patients per 100,000 children per year). Of these, two (6.1%) had positive genetic testing and were defined as familial HLH patients (0.04 familial HLH cases per 100,000 children per year), 28 (84.8%) had evidence of acquired HLH (0.56 acquired HLH cases per 100,000 per year), and 3 (9.1%) had no identified etiology of HLH. Etiologies associated with acquired HLH patients were DENV (n = 22; 84.6%), herpes simplex virus (HSV) (n = 2; 7.7%), systemic-onset juvenile rheumatoid arthritis (n = 2; 7.7%), EBV (n = 2; 3.8%), respiratory syncytial virus (RSV) (n = 1; 3.8%), and coxsackievirus (n = 1; 3.8%). Five (17.9%) acquired HLH cases had evidence of infection with two pathogens, including DENV/HSV (n = 2), DENV/RSV (n = 1), DENV/EBV (n = 1), and EBV/coxsackievirus (n = 1).

### Dengue-associated HLH patients

Of the 22 identified dengue-associated HLH patients, median age was 1 year (range: 0.2–17 years) and 12 (55%) were male (Table 1). Median duration of fever was 8 days (range: 6–25), and median duration of hospitalization was 18.5 days (range: 8–71). Serum ferritin had been quantitated in all 22 patients, and median maximum value was 18,789 μg/L (range: 754–522,000). No significant associations were observed between highest measured ferritin levels and age, sex, length of hospital stay, or total days of fever. The presence of hemophagocytosis was evaluated in bone marrow biopsy or aspirate in 14 of the 22 dengue-associated HLH patients, and was observed in eight (57.1%). Upon discharge, 13 (59.1%) dengue-associated HLH patients were diagnosed with “hemophagocytic lymphohistiocytosis” or “hemophagocytic syndrome”, and three (13.6%) were diagnosed with “macrophage activation syndrome”.

**Table 1:** Demographic characteristics and clinical and laboratory features of 22 patients with dengue-associated hemophagocytic lymphohistiocytosis that were identified at four referral hospitals in Puerto Rico during January 2008 through June 2013.

Case no.	Age (years)	Sex	Duration of fever (days)	Duration of hospitalization (days)	Method of dengue diagnosis	Infecting DENV	Highest measured serum ferritin (μg/L)	Hemophagocytosis seen in bone marrow?
1	0.5	M	8	57	rRT-PCR, MAC ELISA	DENV-1	8,266	Yes
2	0.8	M	5	70	rRT-PCR, MAC ELISA	DENV-1	22,203	Yes
3	0.9	M	5	18	rRT-PCR	DENV-1	19,994	NP
4	0.9	F	7	18	rRT-PCR, MAC ELISA	DENV-1	38,920	No
5	1.1	F	11	19	rRT-PCR	DENV-1	12,269	Yes
6	9	F	12	63	rRT-PCR	DENV-1	53,158	Yes
7	15	M	15	11	rRT-PCR	DENV-1	16,800	NP
8	17	M	3	12	rRT-PCR	DENV-1	60,300	NP
9	17	F	10	15	rRT-PCR	DENV-1	5,210	NP
10	0.2	F	7	10	rRT-PCR, MAC ELISA	DENV-4	9,899	NP
11	0.2	M	6	20	rRT-PCR, MAC ELISA	DENV-4	522,000	No
12	0.4	M	8	60	rRT-PCR, MAC ELISA	DENV-4	27,861	NP
13	0.6	F	6	8	rRT-PCR	DENV-4	44,202	Yes
14	0.6	F	6	17	rRT-PCR	DENV-4	18,789	No
15	7.9	F	8	40	rRT-PCR, MAC ELISA	DENV-4	9,076	No
16	12	F	10	11	rRT-PCR	DENV-4	9,270	NP
17	15	F	9	30	rRT-PCR, MAC ELISA	DENV-4	49,400	Yes
18	0.2	M	7	9	MAC ELISA	UNK	6, 526	No
19	0.6	M	10	10	MAC ELISA	UNK	4,834	NP
20	16	F	11	27	MAC ELISA	UNK	754	No
21	16	M	13	29	MAC ELISA	UNK	16,500	Yes
22	17	M	25	71	MAC ELISA	UNK	77,377	Yes

Abbreviations: DENV = dengue virus; F = female; M = male; rRT-PCR = real-time reverse transcriptase-polymerase chain reaction; MAC ELISA = IgM antibody capture enzyme linked immunosorbent assay; UNK = unknown; NP = not performed

**Fig 1:**
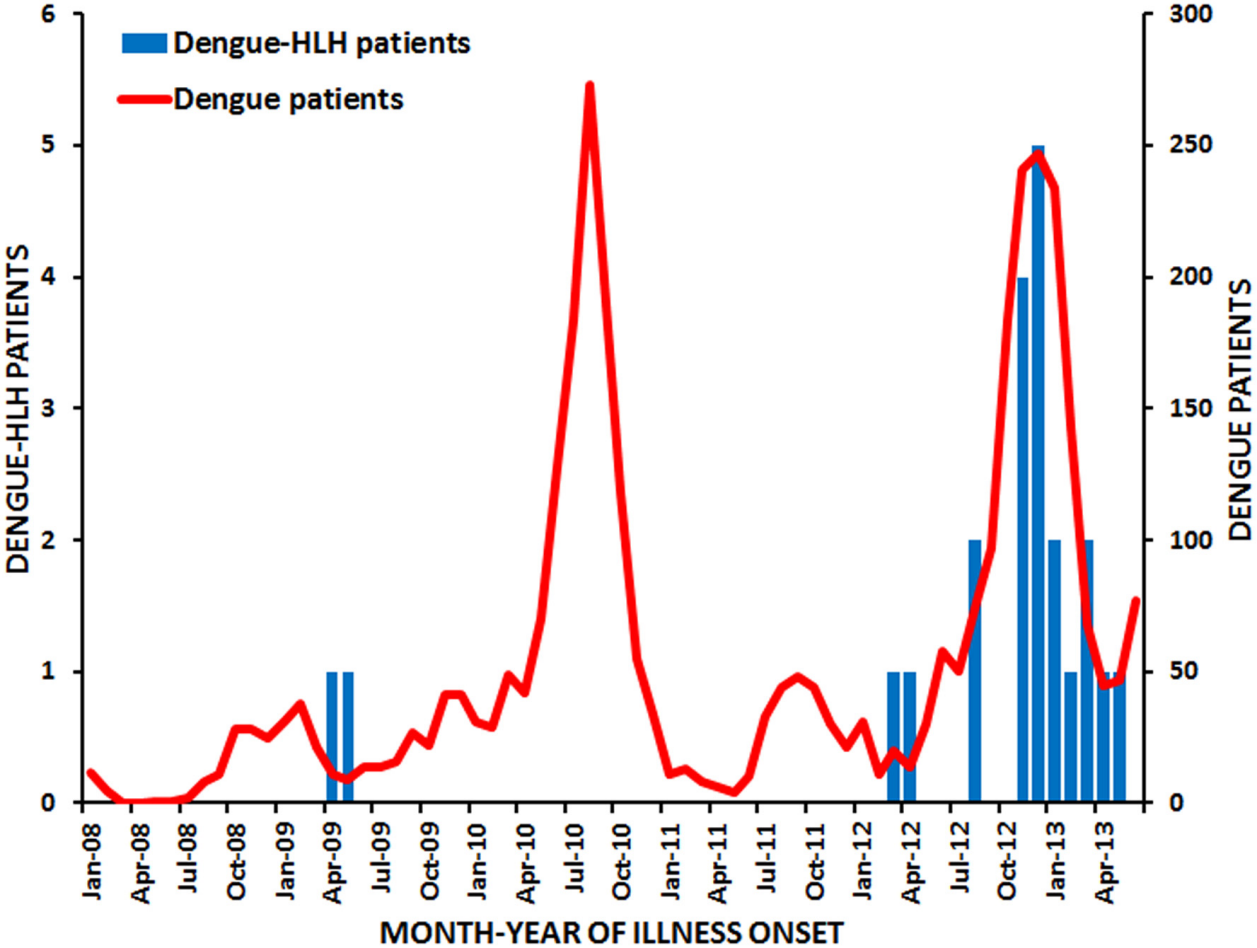
Laboratory-positive dengue (n = 3,475) and dengue-associated hemophagocytic lymphohistiocytosis (HLH) (n = 22) patients identified at four referral hospitals in Puerto Rico during January 2008 through June 2013.

**Fig 2:**
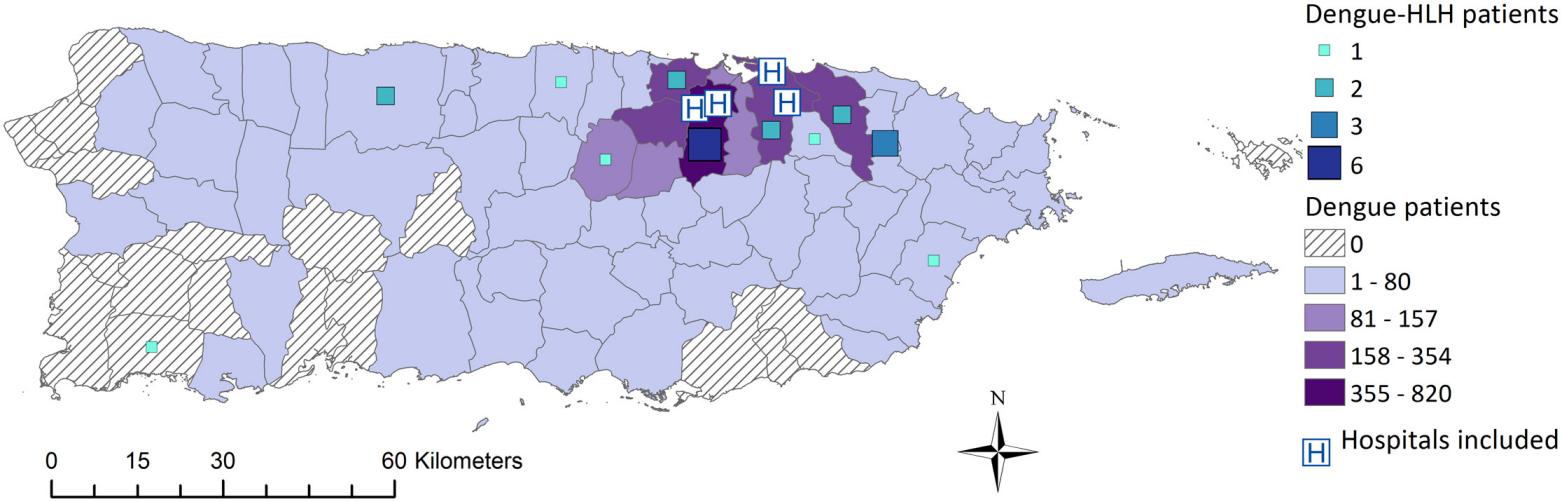
Municipality of residence of dengue and dengue-associated hemophagocytic lymphohistiocytosis patients identified at four referral hospitals in, Puerto Rico, January 2008–June 2013.

Incidence of dengue-associated HLH during January 2008 through June 2013 was 0.44 cases per 100,000 children per year, and was nearly 25-fold higher during 2012–2013 (1.48) than 2008–2011 (0.06). The first two identified dengue-associated HLH patients had illness onset in mid-2009 when incidence of dengue was comparatively low (Fig 1), and the dominant DENVs in circulation were DENV-1 (79%), DENV-2 (17%), and DENV-4 (4%). No dengue-associated HLH patients were identified during the 2010 dengue epidemic that was caused by DENV-1 (69%), DENV-4 (24%), DENV-2 (7%), and DENV-3 (<0.1%) [38]. The large majority (91%) of dengue-associated HLH patients had illness onset during the 2012–2013 dengue epidemic, in which the dominant DENVs were DENV-1 (75%), DENV-4 (25%), DENV-2 (0.4%), and DENV-3 (<0.1%). All but one (95%) dengue-associated HLH patients resided in the San Juan metropolitan area, as did nearly all (98%) dengue patients seen at the same four hospitals over the same time frame (Fig 2). Small sample size of dengue-associated HLH patients precluded analysis of expected incidence of HLH cases in association with dengue cases by municipality of residence.

Of 17 dengue-associated HLH cases in which the infecting DENV was identified by RT-PCR, nine (53%) were DENV-1 and eight (47%) were DENV-4. A moderate correlation existed between monthly incidence of acquired dengue-associated HLH cases and incidence of dengue cases (Pearson coefficient = 0.62, p < 0.0001). A moderate correlation was also found between acquired HLH and number of monthly dengue cases positive by rRT-PCR for either DENV-1 (Pearson coefficient = 0.41; 95% confidence interval [CI]: 0.19–0.60) or DENV-4 (Pearson coefficient = 0.29; 95% CI 0.05–0.50), but not for DENV-2 or DENV-3. Median age of all laboratory-positive dengue cases and most-affected age group was the same during the 2010 and 2012–2013 epidemics (18 years and 10–19 year-olds, respectively).

### Factors associated with development of dengue-associated HLH

Dengue-associated HLH patients (“cases”) were matched 1:4 with randomly selected, laboratory-positive dengue patients (“controls”) by site of hospitalization and month of illness onset. Cases were significantly younger than controls (1 vs. 13 years of age; p = 0.01), and more frequently had evidence of both co-infection (18.2% vs. 4.5%; p = 0.04) as well as recent influenza-like illness (54.5% vs. 25.0%; p = 0.01) (Table 2). No cases or controls received immunosuppressive therapy prior to admission. The mother of one case had an allergic disease (i.e., bronchial asthma), the mother of one control was HIV seropositive, and one control had been born premature. Frequency of chronic medical conditions and taking daily medications did not differ between cases and controls, nor did frequency of taking aspirin or acetaminophen. Frequency of infecting DENV did not differ between cases and controls. Cases had significantly longer duration of fever than controls (7 vs. 5 days, p < 0.01). Cases were hospitalized for longer than controls (18 vs. 5 days, p < 0.01) and were more frequently admitted to the pediatric intensive care unit (100% vs. 15.9%, p < 0.01). One case died (CFR = 4.5%), whereas no controls died. Interventions significantly associated with cases included being intubated, and receiving a blood transfusion, corticosteroids, IVIG, etoposide, or chemotherapy (p < 0.01). No cases or controls were given cyclosporine or received a hematopoietic stem cell transplant.

**Table 2:** Demographic, clinical, and laboratory characteristics of patients with dengue-associated hemophagocytic lymphohistiocytosis (cases) or dengue only (controls) identified in four referral hospitals in Puerto Rico during January 2008 through June 2013.

Characteristic	Cases (N = 22)		Controls (N = 88)		P-value
Age in years, median (range)	1	(0.2–17.9)	13	(0.2–20.4)	0.01
Male, no. (%)	11	(50.0)	55	(62.5)	0.28
Premature birth, no. (%)	3	(13.6)	3	(3.4)	0.08
History of chronic medical condition, no. (%)	10	(45.5)	28	(31.8)	0.30
**Recent exposures, no. (%)**					
Acetaminophen use before hospitalization	18	(81.8)	73	(83.0)	0.89
Excessive* acetaminophen use	4	(18.2)	27	(30.7)	0.26
Antibiotic use before hospitalization	1	(4.5)	3	(3.4)	0.8
Co-infection	4	(18.2)	4	(4.5)	0.04
Recent influenza-like Illness	12	(54.5)	22	(25.0)	0.01
**Clinical characteristics**					
Infecting DENV, no. (%)					
DENV-1	9	(52.9)^†^	41	(71.9)	0.15
DENV-4	8	(47.1) ^†^	16	(28.1)	0.15
Day of illness at presentation, median (range)	3	(0–16)	3	(0–6)	0.29
Length of hospital stay, median days (range)	18	(6–70)	5	(5–15)	<0.01
Admitted to pediatric ICU, no. (%)	22	(100)	14	(15.9)	<0.01
Death, no. (%)	1	(4.5)	0	(0)	0.20
Duration of fever, median days (range)	7	(2–25)	5	(1–9)	<0.01
**Medical interventions, no. (%)**					
Intubated	9	(40.9)	1	(1.1)	<0.01
Blood product transfusion**	18	(81.8)	1	(1.1)	<0.01
Corticosteroids	16	(72.7)	6	(6.8)	<0.01
Intravenous immunoglobulin	13	(59.1)	1	(1.1)	<0.01
Etoposide	8	(36.4)	0	(0)	<0.01
Chemotherapy	8	(36.4)	0	(0)	<0.01
**Signs and symptoms, no. (%)**					
Fever	20	(90.9)	84	(95.5)	0.35
Splenomegaly	17	(77.3)	3	(3.4)	<0.01
Hepatomegaly	19	(86.4)	5	(5.7)	<0.01
Lymphadenopathy	4	(18.2)	4	(4.5)	0.02
Rash	13	(59.1)	34	(38.6)	0.09
**Clinical syndrome, no. (%)**					
Dengue warning signs	22	(100)	65	(73.9)	<0.01
Dengue hemorrhagic fever	13	(59.1)	13	(14.8)	<0.01
Severe dengue	18	(81.8)	13	(14.8)	<0.01
**Clinical laboratory tests, no. (%)**					
Anemia	16	(72.7)	2	(2.3)	<0.01
Thrombocytopenia	20	(90.9)	74	(84.1)	0.41
Leukopenia	17	(77.3)	73	(83.0)	<0.01
Neutropenia	14	(63.6)	45	(51.1)	0.11
Alanine transaminase ≥1,000 IU/L	4	(18.2)	2	(2.3)	0.01
Aspartate transaminase ≥1,000 IU/L	10	(45.5)	7	(8.0)	<0.01
Serum ferritin ≥500 μg/L	17	(77.3)	49	(55.7)	0.02
**Other laboratory tests**^††^					
Serum ferritin ≥ 500 μg/L, no. (%)	13	(86.7)	56	(62.9)	NA
Serum IL-2R ≥ 2400 units/mL, no. (%)	14	(93.3)	77	(90.6)	NA

*>1,000 milligrams (mg)/4 hours (hrs) for individuals aged >12; >320 mg/4hrs for individuals aged 6–12 years, >160 mg/4 hrs for individuals aged 3–6 years, >200 mg/4 hrs for individuals aged 1–3 years, and >90 mg/4 hours for individuals

**transfusion of fresh frozen plasma, packed red blood cells, platelets, or whole blood

^†^denominator is all PCR-positive specimens (n = 17)

^††^Testing done at the CDC-DB with 15 cases and 85 controls that had available serum specimen.

NA = not applicable; raw data not available for analysis

Signs and symptoms significantly associated with cases included splenomegaly, hepatomegaly and lymphadenopathy (p ≤ 0.02). Cases more frequently had warning signs of severe dengue and met the case definitions for either DHF or severe dengue (p < 0.01). Clinical laboratory values significantly associated with cases included anemia, and elevated aminotransferases, aspartate transaminase (AST) and/or alanine transaminase (ALT) (*p* ≤ 0.01). Maximum serum ferritin levels measured while patients were hospitalized were higher in cases (median = 17,794 μg/L; range = 754–522,000) than controls (median = 4,139 μg/L; range = 42–30,346). Serum ferritin level ≥500 μg/L as were more common in cases than in controls (p = 0.02). Using stored serum specimens that had been sent for dengue diagnostic testing, quantitated serum ferritin was more frequently ≥500 μg/L in cases as compared to controls, whereas IL-2Rc was frequently ≥2,400 units/mL in both cases and controls.

## Discussion

During this cluster of dengue-associated HLH cases, infants were most frequently affected and cases were associated with higher morbidity (100% ICU admission and longer length of stay) and mortality (4.5%) than hospitalized dengue patients. As compared to dengue patients, the relationship between dengue-associated HLH patients and hepatomegaly, splenomegaly, anemia, and elevated aminotransferases was expected, since these aspects are clinically consistent with HLH but not necessarily dengue. Conversely, thrombocytopenia and neutropenia were not more likely to be associated with dengue-associated HLH patients, which may be reflective of the similarity of these clinical characteristics with dengue. Last, although morbidity was higher in dengue-associated HLH patients than those hospitalized due to dengue alone, this was not unexpected since several clinical characteristics of HLH overlap with those of severe dengue (e.g., hemorrhage, fluid accumulation, severe liver impairment).

Additionally it is important to note that serum ferritin is a marker of macrophage activation *in vivo*, and is often elevated in dengue patients [7,8]. Level of serum ferritin in often higher in pediatric dengue patients as compared to adults [43]. However, elevated serum ferritin is also a diagnostic criterium for HLH. The significance and role for elevated serum ferritin in dengue-associated HLH patients are therefore unclear, as several such patients in this investigation had either severe dengue or warning signs for progression to severe dengue.

Several possible explanations for this cluster of dengue-associated HLH were explored. An interesting observation was that the majority of dengue-associated HLH patients were infants. Familial HLH frequently presents in the first few years of life, often in association with an infectious agent. Although not all dengue-associated HLH patients were tested for genetic predisposition to HLH, those that were tested were negative. Thus, this outbreak could not be explained by heredity alone. Infants being more frequently identified as dengue-associated HLH patients could be related to increased viremia, heightened immune response to DENV infection, and/or antibody dependent enhancement of disease, the latter of which is associated with waning maternal anti-DENV IgG antibody [5]. Because sufficient volume of serum specimens was unavailable to define dengue-associated HLH patients’ anti-DENV neutralizing antibody profile, we were unable to determine if development of HLH was associated with infection with a specific DENV on the background of a certain immunologic neutralizing antibody combination. However, since the DENVs with which both infants and older children with dengue-associated HLH were infected was not appreciably different from those infecting controls or the distribution of the DENVs circulating in 2012–2013, this possibility would seem unlikely. Nonetheless, we were unable to rule out a role for previous DENV infection affecting the likelihood of infant or other dengue patients progressing to develop HLH. Hence, future investigations should explore the possibility of prior DENV infections and the order in which they occurred as playing a role in progression from dengue to HLH. Conversely, patients with dengue-associated HLH were more likely to be currently or recently infected with a pathogen in addition to DENV, which may lend further support to the idea that immunologic over-stimulation is associated with development of HLH. However, this phenomenon was observed in less than one-fifth of dengue-associated HLH cases, and thus cannot entirely explain the increased incidence of HLH during the 2012–2013 dengue epidemic. Though limited in breadth due to small sample size, we also saw no association between development to dengue-associated HLH and chronic medical conditions, concomitant medications, recent vaccination, and municipality of residence. Last, an island wide evaluation of 1500 dengue inpatient records found a significant reduction in corticosteroid use from 2008–2009 to 2011 that was thought to be due, in large part, to a 2010 CDC training initiative on dengue clinical management (CDC data, in press). This reduction in the use of corticosteroids could have unmasked HLH patients that would have been inadvertently treated prior to this initiative. Nonetheless, although individual explanations may explain some of the identified dengue-associated HLH patients, the reason(s) for increased incidence of HLH specifically with the 2012–2013 dengue epidemic remains unclear.

Few studies have identified the population-specific incidence of HLH, most of which focused on familial HLH in pediatric populations. Such studies have identified 0.12–0.15 HLH cases per 100,000 children per year in Sweden [44], 1 HLH case per 100,000 children per year in Texas [2], and 1 HLH case per 800,000 individuals per year in Japan [45]. Therefore, the incidence of familial HLH identified in this investigation (0.04 per 100,000 children per year) is several fold lower than that observed in previous studies. This difference may be attributable to lack of diagnostic testing for suspected familial HLH cases, clinical under recognition of familial HLH, or potential differences in genetic predisposition to familial HLH. Importantly, the incidence of familial HLH has not been quantitated in African or Hispanic populations.

Of note, the incidence of acquired HLH identified in this investigation (0.56 cases per 100,000 children per year) was 14-fold higher than the incidence of familial HLH. This difference is on par with previous studies in Germany, wherein acquired HLH was roughly 6-fold more common than familial HLH (G. Janka, personal communication). Although pathogens identified to be associated with acquired HLH in this investigation included EBV and other herpesviruses that have been previously associated with HLH, it was striking that nearly four-fifths of acquired HLH cases were associated with DENV infection. Therefore, the comparatively high incidence of HLH identified in this investigation (0.66 HLH cases per 100,000 children per year) was attributable in large part to dengue-associated HLH. However, it is interesting to note that the population-specific incidence of HLH has not been reported from dengue-endemic regions of the tropics. Additional investigation of HLH, and particularly acquired HLH, should be conducted in the tropics to better understand the incidence of HLH and the mechanisms by which DENV and other pathogens trigger HLH.

A prominent strength of this investigation was identification of HLH cases from pediatric referral hospitals, where HLH cases were likely to have been hospitalized. Therefore, it is unlikely than many additional HLH cases were not identified in this population. Conversely, limitations of this investigation include the inability to compare serum ferritin, IL-2 receptor, and natural killer (NK) cell activity–which are well-established markers of HLH–between dengue-associated HLH cases and dengue patients that did not develop HLH. This limitation was attributable to lack of available data for controls (serum ferritin) and infrequency of the tests being requested for both cases and controls (IL-2Rc and NK cell activity). Also, because only municipality of residence was available for most dengue-associated HLH and dengue patients, we were unable to perform a more exact association of patients’ location of residence and consequent factors associated with socio-economic status with development of dengue-associated HLH. Thus, potential environmental or residential exposures were unable to be confidently ruled out. Additionally familial HLH exists as multiple genetic subtypes and genetic tests were not completed for all cases, therefore it is possible that some HLH cases in this investigation were misclassified.

It remains unclear if the increasing incidence of dengue-associated HLH since it was first documented in 1965 is attributable to improved clinical awareness, environmental, genetic, viral, other changes, or some combination thereof. While additional clinical and epidemiologic investigation is conducted in areas with endemic dengue, clinicians seeing patients at risk for dengue should consider HLH in patients with persistent fever, pancytopenia, and multi-organ dysfunction. Previously established protocols for managements of HLH in patients with dengue should be considered [1], including administration of high dose corticosteroids, IVIG, and cyclosporine with or without etoposide.

## Supporting Information

S1 Dataset Case control investigation data. (XLSX)

S1 Checklist STROBE checklist. (DOCX)
